# GNA13 as a prognostic factor and mediator of gastric cancer progression

**DOI:** 10.18632/oncotarget.6780

**Published:** 2015-12-28

**Authors:** Jia-Xing Zhang, Miao Yun, Yi Xu, Jie-Wei Chen, Hui-Wen Weng, Zou-San Zheng, Cui Chen, Dan Xie, Sheng Ye

**Affiliations:** ^1^ Department of Oncology, the First Affiliated Hospital, Sun Yat-Sen University, Guangzhou, PR China; ^2^ Sun Yat-Sen University Cancer Center, State Key Laboratory of Oncology in South China, Collaborative Innovation Center for Cancer Medicine, Guangzhou, PR China; ^3^ Department of Ultrasound, Cancer Center, Sun Yat-Sen University, Guangzhou, PR China; ^4^ Department of Pathology, Cancer Center, Sun Yat-Sen University, Guangzhou, PR China

**Keywords:** GNA13, gastric cancer, proliferation, tumorigenicity

## Abstract

Guanine nucleotide binding protein (G protein), alpha 13 (GNA13) has been implicated as an oncogenic protein in several human cancers. In this study, GNA13 was characterized for its role in gastric cancer (GC) progression and underlying molecular mechanisms. The expression dynamics of GNA13 were examined by immunohistochemistry (IHC) in two independent cohorts of GC samples. A series of *in-vivo* and *in-vitro* assays was performed to elucidate the function of GNA13 in GC and its underlying mechanisms. In both two cohorts of GC samples, we observed that GNA13 was markedly overexpressed in GC tissues and associated closely with aggressive magnitude of GC progression and poor patients' survival. Further study showed that upregulation of GNA13 expression increased the proliferation and tumorigenicity of GC cells *in vitro* and *in vivo*, by promoting cell growth rate, colony formation, and tumor formation in nude mice. By contrast, knockdown of GNA13 effectively suppressed the proliferation and tumorigenicity of GC cells *in vitro* and *in vivo*. Our results also demonstrated that the molecular mechanisms of the effect of GNA13 in GC included promotion of G1/S cell cycle transition through upregulation of c-Myc, activation of AKT and ERK activity, suppression of FOXO1 activity, upregulation of cyclin-dependent kinase (CDK) regulator cyclin D1 and downregulation of CDK inhibitor p21Cip1 and p27Kip1. Our present study illustrated that GNA13 has an important role in promoting proliferation and tumorigenicity of GC, and may represent a novel prognostic biomarker and therapeutic target for this disease.

## INTRODUCTION

Gastric cancer (GC) is one of the most common malignancies and is the second leading cause of cancer-related death worldwide [1]. Although much progress has been made in the diagnosis and treatment of GC, the prognosis of GC patients has remained unsatisfactory, mainly owing to the advanced stage at initial diagnosis and the lack of effective therapies [2, 3]. Accumulating evidence indicates that altered expression of oncogenes and tumor suppressors is associated with the development and progression of GC [4–6]. Therefore, a better understanding of the underlying molecular mechanisms may lead to the development of novel therapeutic approaches [7, 8].

The G protein coupled receptors (GPCRs) are one of the most important classes of cell surface receptor and are involved in the regulation of various cellular processes [9, 10]. GPCRs signal primarily through heterotrimeric G proteins; and among the different types of G proteins, GNA12 and GNA13 have been particularly associated with tumor progression. Most studies have focused on the role of GNA12 in cancer biology; however, few studies have reported the specific role of GNA13 [11–13]. Recently, Li reported a critical role for GNA13 in lysophosphatidic acid (LPA)-stimulated invasive migration of pancreatic cancer cells [14]. GNA13 overexpression also drives an aggressive phenotype in human small cell lung cancer and prostate cancer cells and enhances mouse xenograft tumor growth *in vivo* [15, 16]. We previously found that GNA13 is an important mediator of the epithelial-mesenchymal transition (EMT) during colorectal cancer metastasis [17]. Additionally, GNA13 regulated angiogenesis through induction of VEGFR2 expression [18]. To date, however, the expression pattern and biological role of GNA13 in GC cells has remained largely unknown.

In this study, we found that GNA13 was markedly overexpressed in GC tissues and closely associated with aggressive GC progression and poor survival outcome and that silencing GNA13 expression dramatically suppressed the proliferation and tumorigenicity of GC cells both *in vitro* and *in vivo*, whereas overexpressing GNA13 had the opposite effect. We also showed that GNA13 promoted G1/S cell cycle transition through upregulation of c-Myc transcriptional activity; suppression of FOXO1 activity; enhanced AKT and ERK activity; upregulation of cyclin-dependent kinase (CDK) regulator cyclin D1; and downregulation of CDK inhibitors p21Cip1 and p27Kip1. Taken together, our studies indicated that GNA13 functioned as an oncoprotein during GC progression and that GNA13 might be a potential target for human GC treatment.

## RESULTS

### GNA13 is up-regulated in GC

**Figure 1:**
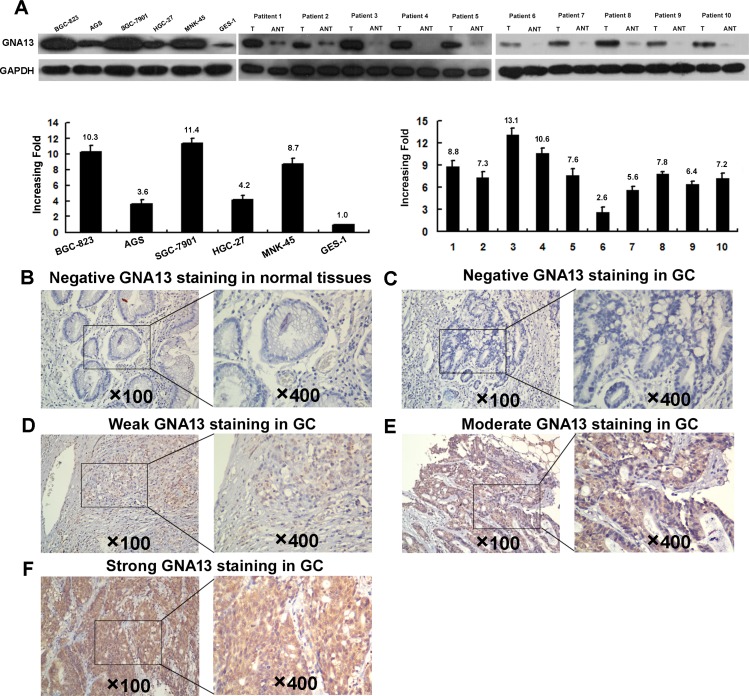
Western blotting, qPCR and IHC assay of the expression pattern of GNA13 in GC tissues and cell lines (**A**) Left panel: Western blotting (upper) and qPCR (lower) assay of GNA13 expression in GSE1 and 5 GC cell lines; Right panle: Western blotting (upper) and qPCR (lower) analysis of GNA13 protein expression in 10 pairs of matched GC tissues (T) and adjacent noncancerous tissues (ANT). GAPDH was used as a loading control. Representative image of negative GNA13 IHC staining (Scoring intensity = 0) (**B**) in normal gastric tissues. Representative images of weak (Scoring intensity = 1) (**C**), moderate (Scoring intensity = 2) (**D**) and strong (Scoring intensity = 3) (**E**) GNA13 IHC staining in GC tissues is shown.

To investigate the expression status of GNA13 in GC, we conducted western blotting and qPCR analysis in five GC cell lines (AGS, BGC-823, HGC-27, MNK-45, and SGC-7901), one immortalized human gastric epithelial mucosa cell line (GES-1), and ten fresh GC tissues (T) with their paired adjacent normal-tissues (ANTs). Interestingly, all five GC cell lines displayed elevated GNA13 mRNA and protein expression compared with GES-1 (Figure 1A: left). Consistently, we found that GNA13 mRNA and protein expression were higher in ten human GC tissues than in the paired ANTs (Figure 1A: right), indicating that GNA13 expression is upregulated in GC.

### Increased GNA13 expression is associated with progression and poor prognosis in GC

**Figure 2:**
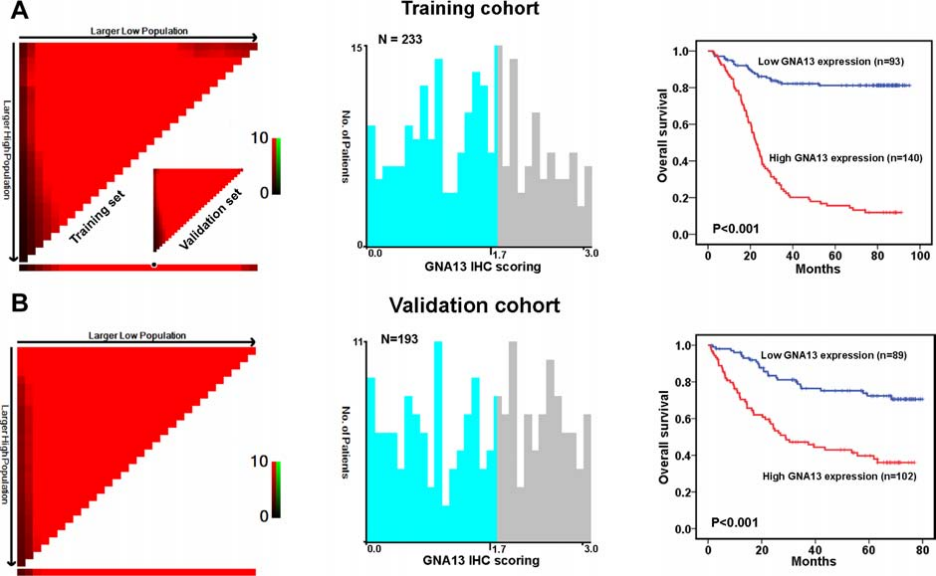
X-tile plots of the prognostic marker of GNA13 in the GC cohorts X-tile analysis was carried out on patient data from the training cohort, equally subdivided into training and validation subsets. X-tile plots of training sets are displayed in the left panels, with matched validation sets in the smaller inset. The plot showed the *χ*^2^ log-rank values created when the cohort was divided into two populations. The cut point was demonstrated on a histogram of the entire cohort (middle panels) and a Kaplan–Meier plot (right panels). *P* values were defined by using the cut point derived from a training subset to parse a separate validation subset. (**A**) GNA13 expression was divided at the optimal cut point, as defined by the most significant on the plot (with positive staining of GNA13; *P* < 0.001). (**B**) The optimal cut point for GNA13 expression determined by X-tile plot of the testing cohort was applied to the validation cohort and reached high statistical significance (*P* < 0.001).

To explore the role of GNA13 in the clinical progression of GC, we examined the expression of GNA13 protein by immunohistochemistry (IHC) in two independent cohorts of GC tissues. Consistently, IHC analysis indicated that GNA13 was markedly upregulated in GC samples (Figure 1B–1E). To achieve statistical significance and avoid arbitrary cut-point selection, we applied the X-tile program to generate optimal cutoff scores. Using X-tile plots for the training cohort, we determined 1.7 as the optimal cut point, and this value was used to divide the cohort into low and high populations (*P* < 0.01, Figure 2A). Applying the cutoff point to the validation cohort also generated highly significant values (*P* < 0.01, Figure 2B). Thus, high GNA13 expression was found in 93 out of 233 (39.9%) GC cases in the training cohort and 90 out of 193 (46.6%) cases in the validation cohort. Quantitative analysis indicated significantly higher GNA13 IHC staining scores in primary tumors than in normal gastric epithelial tissues, with increased IHC scoring in tumors of higher clinical stage ( *P* < 0.05, Supplementary Figure S1). High GNA13 expression was also strongly correlated with clinical stage, T status, N status, and tumor size in two GC cohorts (*P* < 0.05, Supplementary Table S1). These data implied that the expression level of GNA13 increases with GC progression.

To evaluate prognostic values of GNA13 expression and clinicopathological features, receiver operating characteristic (ROC) curves were plotted to test patient survival status. ROC curve analysis confirmed the predictive value of GNA13 regarding overall survival (OS) in the training cohort (area under the curve [AUC] = 0.733, Supplementary Figure S2A). In the validation cohort, GNA13 was also found to be a promising predictor for survival status (AUC =0.719, Supplementary Figure S2B). Furthermore, our univariate and multivariate analyses showed that high GNA13 expression was an independent risk factor for adverse OS in the training cohort (hazard ratio (HR): 8.244; 95% confidence interval (CI): 2.495–9.510, *P* < 0.001; Table 1) and in the validation cohort (HR: 3.135, 95% CI: 1.819–5.401, *P* < 0.001, Table 1). Additionally, survival analysis showed that GNA13 expression could significantly stratify OS in a subset of GC patients with different age, gender, T status, N status, M status, overall clinical stage, tumor grade and tumor size (*P* < 0.05, Supplementary Figure S3).

**Table 1:** Univariate and multivariate analysis of GNA13 expression and various clinicopathological parameters in training and validation cohort patients with G

Variables	Training cohort				Validation cohort			
	Case	HR	(95% CI)	*P* value	Case	HR	(95% CI)	*P* value
**Univariate analysis**								
Age								
< 60yr	149	1			113	1		
≥ 60yr	84	1.47	0.998–2.166	0.051	80	1.259	0.805–1.968	0.313
Gender								
Male	152	1			127	1		
Female	81	1.057	0.708–1.578	0.785	66	1.230	0.780–1.942	0.373
T status								
T1/2	47	1			45	1		
T3/4	186	8.838	3.250–24.036	< 0.001	148	3.351	1.610–6.973	0.001
N								
N0	65	1			63	1		
N1/2	168	5.034	2.621–9.667	< 0.001	130	6.309	3.027–13.150	< 0.001
M								
M0	213	1			159	1		
M1	20	3.93	2.316–6.667	< 0.001	34	3.468	2.128–5.652	< 0.001
Clinical stage								
I/II	77	1			70	1		
III/IV	156	7.012	3.648–13.476	< 0.001	123	6.511	3.240–13.082	< 0.001
Grade								
G1/2	69	1			47	1		
G3	164	1.676	1.055–2.662	0.029	146	2.252	1.216–4.170	0.010
Tumor size								
< 4 cm	120	1			124	1		
≥ 4 cm	113	1.845	1.249–2.726	0.002	69	1.689	1.079–2.644	0.022
Therapy								
Surgery only	108	1			84			
Surgery + CT	125	1.378	0.931–2.040	0.109	109	0.899	0.566–1.429	0.654
Ki-67 expression								
< 50%	135	1			116	1		
≥ 50%	98	4.137	2.734–6.261	< 0.001	77	1.629	1.044–2.541	0.032
GNA13 expression								
Low expression	140	1			103	1		
High expression	93	8.244	5.219–13.023	< 0.001	90	3.176	1.979–5.096	< 0.001
**Multivariate analysis**								
T status								
T1/2	47	1			45	1		
T3/4	186	3.411	1.207–9.642	0.021	148	1.544	0.711–3.356	0.272
N								
N0	65	1			63	1		
N1/2	168	2.137	1.070–4.270	0.031	130	4.021	1.878–8.608	< 0.001
M								
M0	213	1			159	1		
M1	20	2.546	1.455–4.455	0.001	34	2.771	1.655–4.639	< 0.001
Grade								
G1/2	69	1			47	1		
G3	164	1.252	0.769–2.036	0.366	146	2.361	1.249–4.465	0.008
Tumor size								
< 4 cm	120	1			124	1		
≥ 4 cm	113	1.297	0.872–1.930	0.199	69	1.097	0.691–1.742	0.695
Ki-67 expression								
< 50%	135	1			116	1		
≥ 50%	98	1.189	0.653–2.165	0.572	77	0.912	0.543–1.533	0.729
GNA13 expression								
Low expression	140	1			103	1		
High expression	93	4.871	2.495–9.510	< 0.001	90	3.135	1.819–5.401	< 0.001

GC, gastric cancer; CT,chemotherapy; Cox propotional hazard regression model, enter; HR, Hazard ratio; CI, confidence interval.

### GNA13 promotes the proliferation of GC cells

**Figure 3:**
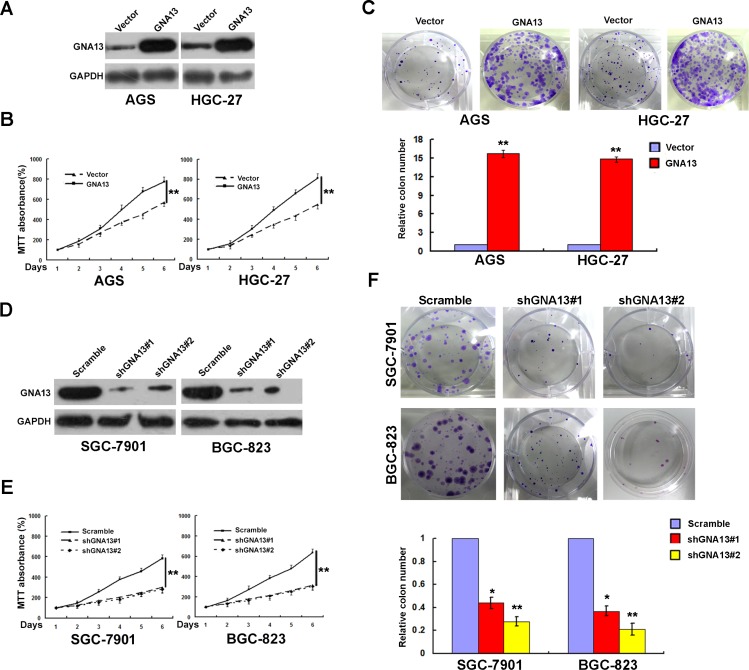
GNA13 promotes human GC cell growth and proliferation *in vitro* (**A**) Ectopic expression of GNA13 in AGS and HGC-27 cells analyzed by western blotting. (**B** and **C**) Ectopic expression of GNA13 promoted proliferation ability of AGS and HGC-27 cell as determined by MTT assays (B) and colony formation assays (C). (**D**) Knockdown of endogenous GNA13 in specific shRNA transduced stable SGC-7901 and BGC-823 cells. (**E** and **F**), knockdown of GNA13 inhibits cell growth as determined by MTT assays (E) and colony formation assays (F). *P < 0.05; **P < 0.01.

To further elucidate the role of GNA13 in GC progression, GNA13 was stably transfected into GC cell lines AGS and HGC-27, which showed endogenous low GNA13 expression (Figure 3A). MTT and colony formation assays showed that the proliferation rate of GNA13-overexpressing cells was significantly higher than in the vector-control cells (Figure 3B–3C). To confirm this result, we knocked down endogenous GNA13 in SGC-7901 and BGC-823 GC cells by expressing short hairpin RNAs (shRNA) (Figure 3D). Consistently, MTT and colony formation assay showed that the proliferation rates were significantly compromised (Figure 3E–3F). These data showed that GNA13 has a critical role in the proliferation of GC cells *in vitro*. We also examined the relationship between GNA13 and Ki-67 expression in GC tissue samples. The tumor samples with high levels of GNA13 staining also exhibited strong Ki-67 staining signals, whereas areas with low GNA13 expression exhibited weak Ki67 expression (Supplementary Figure S4). Chi-square testing also indicated a significant correlation between GNA13 expression and the Ki-67 labeling index in GC (*P* < 0.001, Supplementary Table S1).

### GNA13 promotes the tumorigenicity of GC cells both *in vitro* and *in vivo*

**Figure 4:**
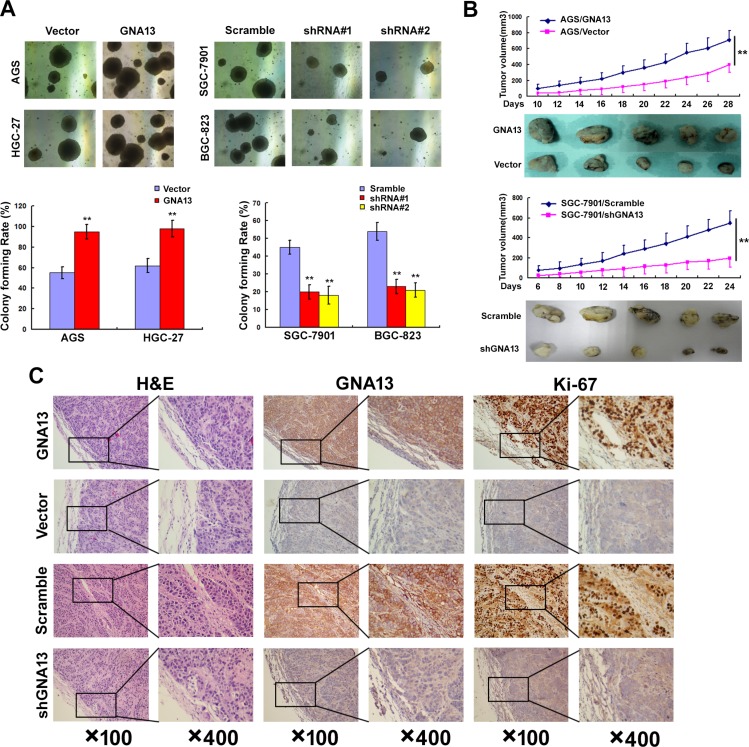
GNA13 promotes the tumorigenicity of GC cells *in vitro* and *in vivo* (**A**) Anchorage-independent growth assay in GNA13-overexpressing cells (Left) and GNA13-silenced cells (Right). Soft agar colony formation (colonies larger than 0.1 mm diameter) was quantified after 14 days of culture (Lower panel). (**B**) AGS/GNA13 and AGS/Vector cells, and SGC-7901/shGNA13 and SGC-7901/scramble cells were injected in the hindlimbs of nude mice (*n* = 5). Tumor volumes were measured on the indicated days. (**C**) Histopathology of xenograft tumors. The tumor sections were under H&E staining and IHC staining using antibodies against GNA13 and Ki-67. *P < 0.05; **P < 0.01.

We next explored the effect of GNA13 on the tumorigenicity of GC cells using an anchorage-independent growth assay. As shown in Figure 4A, upregulation of GNA13 dramatically increased colony number and colony size on soft agar, whereas downregulation led to a decrease. To confirm this effect *in vivo*, AGS/GNA13, AGS/Vector, SGC-7901/shGNA13 and SGC-7901/Scramble cells were inoculated in nude mice. Similarly, AGS/GNA13 tumors grew significantly faster than control vector tumors, whereas the tumors formed by SGC-7901/shGNA13 cells grew at a much slower rate than control SGC-7901/Scramble tumors (Figure 4B). Consistently, our IHC analysis showed that tumors formed by AGS/GNA13 cells displayed much stronger GNA13 staining and higher Ki-67 indices compared to tumors formed by AGS/Vector cells, whereas tumors formed by SGC-7901/shGNA13 cells exhibited significant inhibition of GNA13 staining and lower Ki-67 indices (Figure 4C). Collectively, these results indicate that GNA13 has an important role in enhancing tumorigenicity of GC cells both *in vitro* and *in vivo*.

### GNA13 accelerates the G1-S phase transition in GC cells

**Figure 5:**
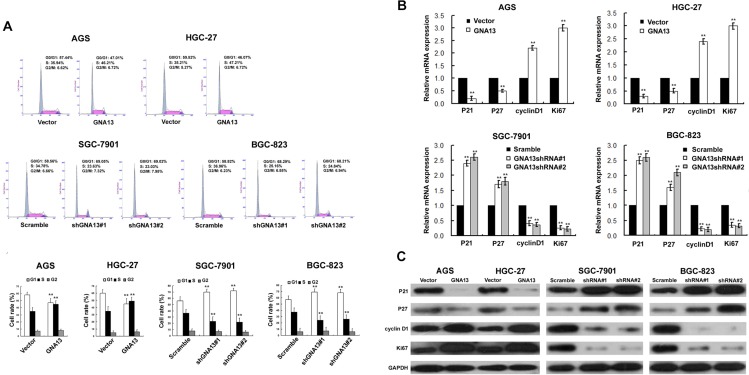
The effect of GNA13 on cell cycle of distribution and G1–S-phase regulators of GC cells (**A**) Upper: representative histograms depicting cell cycle profiles of indicated cells. Cells were stained with PI and analyzed by flow cytometry. Lower: proportion of cells in various phases of the cell cycle. (**B**) Real-time PCR analysis of p21Cip1, p27Kip1, Ki67, and cyclinD1 mRNA expression in GNA13-transduced cells (upper panel) or GNA13 shRNA–infected cells (lower panel). Expression levels were normalized to GAPDH. (**C**) Western blot analysis of p21Cip1, p27Kip1, cyclin D1, and Ki67 proteins in GNA13-transduced cells or GNA13 shRNA–infected cells. GAPDH was used as a loading control. *P < 0.05; **P < 0.01.

To understand the mechanism by which GNA13 promotes proliferation of GC cells, we performed flow cytometry analysis. As shown in Figure 5A–5B, overexpressing GNA13 significantly decreased the proportion of cells in the G0/G1 phase and increased those in the S phase, but silencing GNA13 reduced the percentage of S phase cells and increased G0/G1 phase cells, suggesting that GNA13 accelerates G1-S phase transition in GC cells.

Since GNA13 expression appeared to be tightly linked to the G1/S phase transition of GC cells, we further investigated whether cell cycle factors, including cyclin D1, and p21Cip1 and p27Kip1, could be regulated by GNA13. Our qRT-PCR and western blot analysis showed a significant upregulation of cyclin D1 and Ki67, accompanied by downregulation of p21Cip1 and p27Kip1 mRNA and protein levels in GNA13-overexpressing cells compared to control cells (Figure 5C–5D). By contrast, p21Cip1 and p27Kip1 were significantly increased in GNA13-silenced cells, whereas cyclin D1 and Ki67 were decreased (Figure 5C–5D).

### GNA13 enhances c-Myc transcriptional activity, suppresses FOXO1 transactivity and activates AKT, ERK signaling pathways

**Figure 6:**
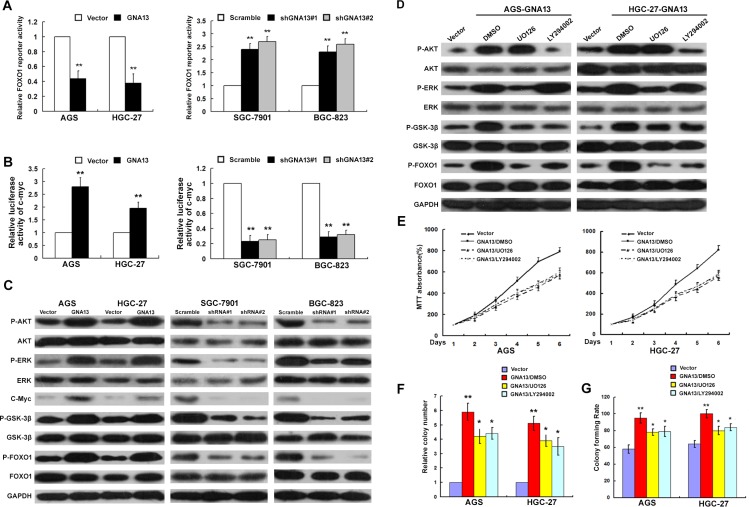
GNA13 downregulates FOXO1 transcriptional activity via activation of the PI3K/AKT and MAPK/ERK signaling pathway (**A** and **B**) Related FOXO1 reporter activity (A) and c-Myc reporter activity (**B**) in GNA13-transduced cells or GNA13 shRNA–infected cells. (**C**), Western blot analysis of p-AKT, total AKT, p-ERK, total ERK, c-Myc, p-GSK-3β, total GSK-3β, p-FOXO1, and total FOXO1 in GNA13-transduced cells or GNA13 shRNA–infected cells. (**D**) AGS/GNA13 and HGC-27/GNA13 cells were treated with the AKT inhibitor LY294002 (20 lM), the ERK kinase inhibitor U0126 (20 lM) or DMSO for 24 h, then harvested to examine the expression levels of the indicated proteins by Western blotting. (**E**, **F** and **G**) AGS/GNA13 and HGC-27/GNA13 proliferation and tumorigenicity were determined by MTT (E), colony formation assays (F) and anchorage-independent growth assay (G) after treatment with LY294002, U0126 or DMSO. *P < 0.05; **P < 0.01.

As FOXO1 and c-Myc transcriptionally regulate p21Cip1, p27Kip1, and cyclin D1 [19, 20], we further investigated whether GNA13 exerted these functions by modulating the transactivity of FOXO1 and c-Myc. As shown in Figure 6A–6B, the transactivity and expression level of FOXO1 and c-Myc significantly decreased in GNA13-overexpressing cells and increased in GNA13-silenced cells. AKT and ERK kinases are known to have key roles in phosphorylating and repressing FOXO1 transcriptional activity [21, 22]. As predicted, phospho-AKT (p-AKT), phosphor-ERK (p-ERK) and phospho-GSK-3β(p-GSK-3β) levels were increased by overexpressing GNA13 but decreased by its silencing, suggesting that GNA13 downregulates FOXO1 transcriptional activity via activation of the PI3K/AKT and MAPK/ERK signaling pathways (Figure 6C). In addition, in the subcutaneous implantation nude mouse models bearing human GC, protein levels of p-AKT and p-ERK in AGS/GNA13 were upregulated. However, in the SGC-7901/shGNA13 group, the expression levels of p-AKT and p-ERK were significantly downregulated (Supplementary Figure S5). In GC samples, p-AKT and p-ERK levels were dramatically increased in high-GNA13 GC tissues compared with low-GNA13 GC tissues (Supplementary Figure S6A). Furthermore, the expression of GNA13 was positively correlated with p-AKT (r = 0.754, *P* < 0.001; r = 0.741, *P* < 0.001) and p-ERK (r = 0.827, *P* < 0.001; r = 0.774, *P* < 0.001) (Supplementary Figure S6B) in both training and validation cohorts. These data indicate that GNA13 exerts its pro-oncogenic function via upregulation of c-Myc transcriptional activity and activation of the PI3K/AKT /FOXO1 and MAPK/ERK /FOXO1 pathways.

To confirm these results, we treated GNA13-overexpressed GC cells with an AKT inhibitor (LY294002) or ERK inhibitor (U0126). As shown in Figure 6D, the expression levels of p-ERK, p-AKT, p-GSK-3β, and p-FOXO1 were significantly reduced by both U0126 and LY294002 in GNA13-overexpressed GC cells. We also examined the growth and tumorigenicity ability of GNA13-overexpressed GC cells using LY294002 or U0126. MTT, colony formation and anchorage-independent growth assays showed that the growth of GNA13-overexpressed cells was significantly compromised by treatment with the AKT or ERK inhibitors compared to control cells (Figure 6E–6G). By contrast, we observed on obvious effect on cell proliferation and colony formation by treatment with the AKT or ERK inhibitors in vector control cells (Supplementary Figure S7). Moreover, colony formation and MTT assays showed that silencing of FOXO1 restored the growth rate of GNA13-silenced GC cells, suggesting that FOXO1 plays an important role in the effect of GNA13 on proliferation in GC cells (Supplementary Figure S8). Taken together, these data indicate that GNA13 may promote proliferation partly via activation of the PI3K/AKT/FOXO1 and MAPK/ERK /FOXO1 signaling pathways.

## DISCUSSION

In this study, we reported for the first time that elevated GNA13 expression in GC is associated with an aggressive phenotype and inferior survival outcomes. *In vitro* and *in vivo* studies demonstrated that GNA13 could promote cell proliferation by accelerating the G1-S-phase transition through upregulation of c-Myc transactivity and downregulation of FOXO1 transcriptional activity via activation of PI3K/AKT and MAPK/ERK signaling. These findings suggest that GNA13 has a vital role in the development and progression of human GC and might serve as a novel therapeutic target.

The development of GC is a multi-step process involving the loss of tumor suppressor genes and activation of oncogenes. At present, most GC-related deaths are due to advanced disease, and diagnosed when metastases have already disseminated to lymph nodes or distant organs. Therefore, it is of great clinical value to identify potential early biomarkers for diagnosis and prognosis. In this study, we observed that upregulation of GNA13 mRNA and protein is a common event in both GC cell lines and human GC tissues. IHC analysis in two large GC samples showed that expression levels of GNA13 appeared to increase with cancer progression: a significant increase in GNA13 expression was observed from normal gastric tissues to early stage GC samples, and from early stage to advanced stage GC samples. High GNA13 expression correlated significantly with aggressive clinical characteristics and poor survival. Notably, we observed a significant correlation between GNA13 expression and proliferation index; GNA13 was strongly expressed in Ki-67 highly-expressed lesions of human GC cells, implying a potential proliferation-promoting role in GCs. Our further *in vivo* and *in vitro* studies implied that GNA13 promotes GC cell proliferation and cell cycle progression, while silencing GNA13 inhibits proliferation and colony formation in human GC cells. Similar results were observed in other human cancers, such as small cell lung cancer and prostate cancer, in which the increased expression of GNA13 was associated with malignant phenotypes or inferior prognosis [15, 16]. Our data, in agreement with previous studies, indicated that GNA13 expression corresponds to the progression of GC and might facilitate its invasive phenotype.

Although the potential oncogenic functions of GNA13 have been implicated in several human malignancies, the precise mechanism remains largely unknown. GNA13 has been shown to be involved in stimulating cell migration modulated by GPCRs, as well as by receptor tyrosine kinases [23–25]. Recently, overexpression of GNA13 has been observed in several human cancers and was associated with cancer development and progression [15, 16]. We previously reported that GNA13 could promote colorectal cancer metastasis by triggering the EMT [17]. Here, we showed that GNA13 could accelerate the G1/S phase transition by regulating expression of cyclin D1, p21Cip1 and p27Kip1. To further investigate the underlying mechanism, we investigated the levels of c-Myc and FOXO1, since cyclin D1, p21Cip1 and p27Kip1 are known downstream targets of c-Myc and FOXO1 [19, 20]. c-Myc, a transcriptional regulator and oncogene, has an essential role in the regulation of many physiological processes, including cell cycle control, apoptosis, protein synthesis, and cell adhesion [26, 27]. By contrast, emerging evidence suggests that FOXO transcription factors function as tumor suppressors by regulating expression genes involved in apoptosis, cell proliferation and genotoxic/oxidative stresses [28, 29]. In the present study, we observed repressed c-Myc and enhanced FOXO1 transactivity in GNA13-silenced GC cells, and increased c-Myc and decreased FOXO1 transactivity in GNA13-transduced GC cells, which was associated with alterations in the expression of cell cycle inhibitors (p21Cip1 and p27Kip1) and the CDK regulators (cyclin D1), suggesting that GNA13-induced proliferation and tumorigenesis might be due to modulation of c-Myc and FOXO1 activity.

Previous studies showed that both the PI3K/AKT and the MAPK/ERK signal transduction cascades, which are required for cell cycle progression through the G1 phase, were frequently involved in promotion of proliferation. It has been shown that activated AKT and ERK inhibited cellular levels of p21Cip1, p27Kip1 and induction of cyclin D1 mRNA and protein, thereby promoting cell proliferation [21, 30–32]. Moreover, activation of AKT and ERK stimulated the phosphorylation of various downstream targets, including GSK-3β, BAD, and the FOXO family of transcription factors. In particular, activated AKT and ERK could result in phosphorylation of FOXO1, which led to downregulation of FOXO1 transactivity via ubiquitin-proteasome-mediated degradation and thus repression of FOXO1-mediated growth arrest [21, 22]. In this study, we observed that levels of phospho-AKT and phospho-ERK, and downstream target proteins phospho-GSK-3β and phospho-FOXO1, were increased in GNA13-overexpressing cells and decreased in GNA13-silenced cells. Moreover, treatment with AKT or ERK inhibitors showed that upregulation of phospho-GSK-3β and phospho-FOXO1 was significantly attenuated in GNA13-overexpressed GC cells, along with significant suppression of cellular growth and colony formation. Thus, we speculate that the modulation of G1-S-phase transition by GNA13, as well as p21Cip1, p27Kip1, and cyclin D1 expression, is probably due to activation of the PI3K/AKT/FOXO3a and MAPK/ERK/FOXO3a pathways. This suggests that GNA13-targeting strategies could potentially be used to deliver an anti-proliferative therapeutic effect through deactivating the PI3K/AKT or MAPK/ERK signaling pathways.

Our present study also suffered several limitations. Of note, the native G-alpha 13 protein needs to be activated to initiate signaling [23]. In this present study, we only overexpressed GNA13 in GC cell lines. To rule out any indirect role of GNA13 in GC progression, the constitutively active form of GNA13 in GC cell lines should be further explored. In contrast to us, Muppidi JR reported GNA13 signaling is frequently disrupted in germinal center B cell-derived lymphoma, and exerts dual actions in suppressing growth and blocking dissemination of germinal center B cells [33]. Thus, these data, combined with our findings, suggested that the function of GNA13 on different type of tumors might have characteristics of tissue-specific.

In summary, we have demonstrated that GNA13 has an important role in human GC progression and provided insights into the underlying mechanisms involved. Furthermore, our results suggest a potential role for GNA13 as a clinical predictor of disease progression, prognosis and survival in GC patients.

## MATERIALS AND METHODS

### Cell lines

Gastric cancer cell lines (AGS, BGC-823, HGC-27, MNK-45, and SGC-7901), and one immortalized human gastric epithelial mucosa cell line (GES-1) were grown in DMEM medium supplemented with 10% fetal bovine serum.

### Samples and patients

We used 426 formalin-fixed paraffin-embedded (FFPE) tumorous and adjacent non-tumorous gastric tissues samples from 426 GC patients in this study. For the training cohort, FFPE samples were obtained from 233 patients with GC disease from Sun Yat-sen University Cancer Center, between January 2007 and December 2007. In parallel, we obtained another independent validation cohort of FFPE samples from 193 GC patients from Sun Yat-sen University Cancer Center, between January 2008 and December 2008. In addition, 10 fresh pairs of tumorous and matched adjacent non-tumorous gastric tissues samples from GC patients who underwent curative surgery in Sun Yat-sen University Cancer Center between January 2014 and May 2014 were frozen and stored in liquid nitrogen until further use. All the samples used in this study contained matched tumors (percentage of tumor cells ≥ 70%) and corresponding normal mucosal tissue (> 5 cm laterally from the edge of the cancerous region); all the patients who had a single primary lesion and no neoadjuvant therapy before operation were included in the study. The patients were followed ever 3 months for the first year and then every 6 months for the next 2 years, and finally annually. The patients who did not have the followed-up information were excluded from this study. The median follow-up time was 57.5 months (range, 5–95 months). The diagnostic examinations consisted of CT, chest X-ray, abdominal ultrasonography and bone scan when necessary to detect recurrence and/or metastasis. The overall survival (OS) was defined as the time from diagnosis to the date of the death date or when censured at the latest date if patients were still alive. The clinicopathologic characteristics of the patients in each cohort are summarized in Supplementary Table S2 and Supplementary Figure S9. Clinical samples used in this study were approved by the Committees for Ethical Review of Research at Sun Yat-Sen University (Guangzhou, China).

### RNA isolation and quantitative real-time PCR

Total RNA was extracted from cultured cells and fresh tissues with TRIzol (Invitrogen, Calsbad, CA) according to the manufacturer's instructions. Real-time PCR was carried out using SYBR Green SuperMix (Roche, Basel, Switzerland) and ABI7900HT Fast Real-Time PCR system (Applied Biosystems, Foster City, CA). Expression data were normalized to the Glyceraldehyde-3-phosphate dehydrogenase (GAPDH). The primer sequences used in the study were listed in Supplementary Table S3.

### Vector construction and retroviral infection

The coding sequences of GNA13 were amplified and cloned into pcDNA3.1 (+) to generate GNA13 expression vector. The primers used are shown in Supplementary Table S1. The sequences of two human short hairpin RNA (shRNA) sequences to repress GNA13 expression are listed as follows: GNA13 shRNA#1: 5′-GCCCAAGGAATGGTGGAAACA-3′; GNA13 shRNA#2: 5′-GGATAACTTGGATAAACTTGG-3′(Genecopoeia, Guangzhou, China). Cells transfected with empty vector were used as controls. The vectors were packaged using the ViraPower Mix (Genecopoeia, Guangzhou, China) in 293FT cells. After culturing for 48 hours, the lentiviral particles in the supernatant were harvested and filtered by centrifugation at 500 *g* for 10 min, and then transfected into the GC cells.

### Luciferase reporter assay

The reporter plasmids for detecting the transcriptional activity of FOXO1 and c-Myc were generated as described previously [34, 35]. The firefly luciferase construct was cotransfected with a control Renilla luciferase vector into GNA13-overexpressing or GNA13-suppressing GC cells. A dual luciferase assay (Promega) was performed 48 h after transfection. The experiments were performed independently in triplicate.

### Colony formation assay

Twenty-four hours after infection, 500 infected cells were placed in a fresh six-well plate and cultured for 2 weeks. Colonies were fixed with methanol and stained with 0.1% crystal violet in 20% methanol for 15 min.

### Western blot (WB) assay

Equal amounts of whole cell and tissue lysates were resolved by SDS-polyacrylamidegel electrophoresis (PAGE) and electrotransferred on a polyvinylidene difluoride (PVDF) membrane (Pall Corp., Port Washington, NY). The following primary antibodies were used: anti-GNA13 (Abcam, Cambridge, UK); anti-GAPDH, anti-GSK3β, anti-p-GSK3β, anti-AKT, anti-p-AKT, anti-ERK, anti-p-ERK, anti-c-Myc, anti-FOXO1, anti-p-FOXO1 (Cell signaling Technology, Beverly, MA).

### Anchorage-independent growth ability assay

Five hundred cells were trypsinized and suspended in 2 ml complete medium plus 0.3% agar (Sigma, Saint Louis, MI). The agar-cell mixture was plated on top of a bottom layer with 1% agar completed medium mixture. About 14 days, viable colonies that were larger than 0.1 mm were counted. The experiment was carried out for each cell line in triplicates.

### Cell cycle analysis

Cells were fixed in 70% ethanol and incubated at −20°C overnight. Cells were then washed twice and resuspended in 500 lL of staining solution (50 lg/mL of propidium iodide, 100 lg/mL RNAase and 0.2% Triton X-100) for 30 min. The fluorescence associated with PI-bound DNA was measured by flow cytometry (Beckman Coulter, cytomics FC 500, CA). The cell cycle profiles, including G1-, S-, and G2/M-phases, were calculated using MultiCycle software.

### Immunohistochemical (IHC) staining and selection the optimal cutoff value

We used the Dako Real Envision Kit (K5007, Dako) for IHC staining analysis, and the staining protocol in this study were described previously. Staining intensity was scored manually by two independent experienced pathologists as 0 = no staining, 1 = weak staining, 2 = moderate staining, and 3=strong staining. Tumor cells in five fields were randomly selected and scored based on the percentage of positively stained cells (0–100%). The final IHC score was calculated by multiplying the intensity score with the percentage of positive cells (range from 0 to 3).

The optimal cutoff score of GNA13 expression was selected using X-tile plots [36]. X-title data were presented in a right triangular grid where each point represents a different cut-point. The intensity of the color of each cutoff point represents the strength of the association. The X-title software allows the user to move a cursor across the grid and provide an “on-the-fly” histogram of the resulting population subsets along with an associated Kaplan–Meier curve. The X-title software provides a method of dividing a single cohort into training and validation subsets for *P* value estimation. In addition, the software can perform standard Monte Carlo simulations (e.g., cross-validation) to produce corrected *P* values to assess statistical significance of data assessed by multiple cut-points. The X-tile program can automatically select the optimal data cut-point according to the highest chi-square value (minimum *P* value) defined by Kaplan–Meier survival analysis and log-rank test [37]. X-tile plots were performed with X-tile software version 3.6.1 (Yale University School of Medicine).

### Xenograft tumor growth assay

Xenograft tumor growth assay was established by subcutaneous injection of AGS/GNA13, SGC-7901/shGNA13 and their respective control cells (2*10^6^) the inguinal folds of 4-week-old severe combined immunodeficient (SCID-Beige) mice, respectively. The mice were monitored daily for palpable tumor formation and tumors were measured using a Vernier caliper, and also weighed and photographed. All animal experiments were conducted according to the institutional standard guidelines at Sun Yat-Sen University.

### Statistical analysis

Statistical analysis was performed using a SPSS software package (SPSS Standard version 16.0, SPSS Inc). For survival analysis, the optimal cutoff point for GNA13 expression was obtained using X-tile software version 3.6.1 as mentioned above (Yale University School of Medicine, New Haven, CT, USA). The statistical significance of the correlation between GNA13 expression level and patient survival was estimated using the Mantel–Cox log-rank test. Monte Carlo simulations were used to adjust for multiple observations in optimal cutoff point selection. Receiver operating characteristic (ROC) curve analysis was conducted to evaluate the predictive value of the parameters. Comparisons between groups for statistical significance were performed with a 2-tailed paired Student's *t* test. Bivariate correlations between study variables were calculated by Pearson's correlation coefficients. Differences between variables were assessed by the Chi-square test or Fisher's exact test. For survival analysis, we analysed all GC patients by Kaplane-Meier analysis. A log rank test was used to compare different survival curves. Multivariate survival analysis was performed on all parameters that were found to be significant in univariate analysis using the Cox regression model. *P* values < 0.05 were considered significant.

## SUPPLEMENTARY MATERIALS FIGURES AND TABLES


