# Supplementary material for: GNA13 as a prognostic factor and mediator of gastric cancer progression
Source: Oncotarget. 2015 Dec 28;7(4):4414–27. doi: 10.18632/oncotarget.6780 (PMC4826215; doi:10.18632/oncotarget.6780)
Supplement: Supplementary file 1 [file oncotarget-07-4414-s001.pdf]

GNA13 as a prognostic factor and mediator of gastric cancer progression

Supplementary Materials

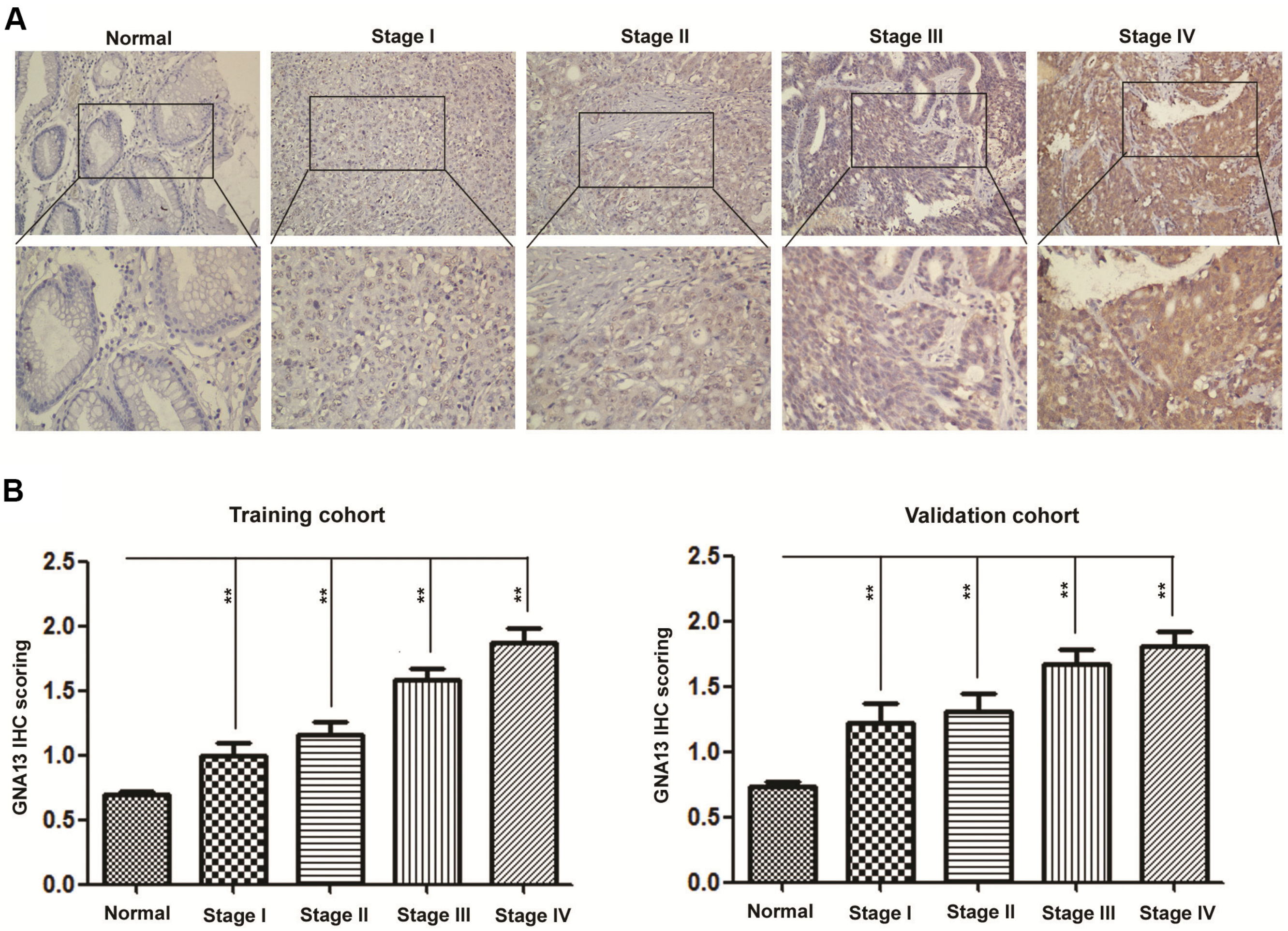

Supplementary Figure S1: GNA13 expression in gastric cancer tissues was positively associated with clinical stage. (**A**) The representative images of GNA13 IHC staining as tumor stage increased. (**B**) The IHC scoring of GNA13 staining increases as tumor stage increased (P< 0.001). *$P < 0.05$; **$P < 0.01$.

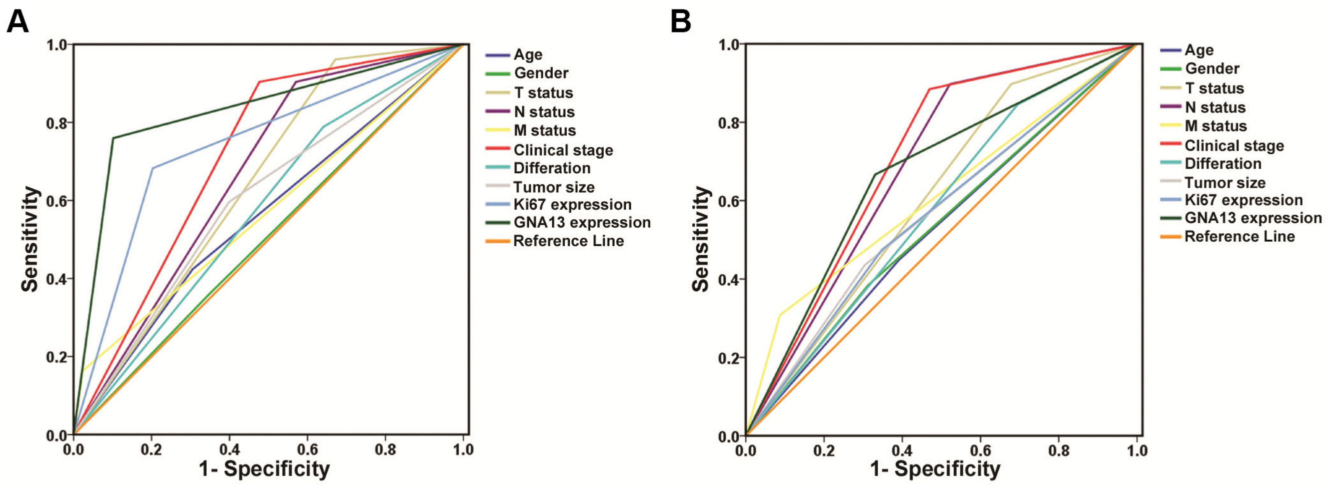

Supplementary Figure S2: ROC curve analysis for different clinicopathological features and GNA13 expression was performed to evaluate the survival status. (**A**) Age (AUC = 0.559; $P = 0.121$), gender (AUC = 0.506; $P = 0.875$), T statue (AUC = 0.645; $P < 0.001$), N statue (AUC = 0.667; $P < 0.001$), M statue (AUC = 0.570; $P = 0.067$), clinical stage (AUC = 0.714; $P < 0.001$), differentiation (AUC = 0.574; $P = 0.053$), tumor size (AUC = 0.599; $P = 0.010$), Ki67 expression (AUC = 0.740; $P < 0.001$), and GNA13 expression (AUC = 0.829; $P < 0.001$) implied statistical associations with survival in the training cohort. (**B**) Age (AUC = 0.529; $P = 0.499$), gender (AUC = 0.536; $P = 0.399$), T statue (AUC = 0.610; $P = 0.010$), N statue (AUC = 0.688; $P < 0.001$), M statue (AUC = 0.610; $P = 0.009$), clinical stage (AUC = 0.708; $P < 0.001$), differentiation (AUC = 0.575; $P = 0.076$), tumor size (AUC = 0.566; $P = 0.121$), Ki67 expression (AUC = 0.563; $P = 0.136$), and GNA13 expression (AUC = 0.668; $P < 0.001$) were used to test the survival status in validation cohort.

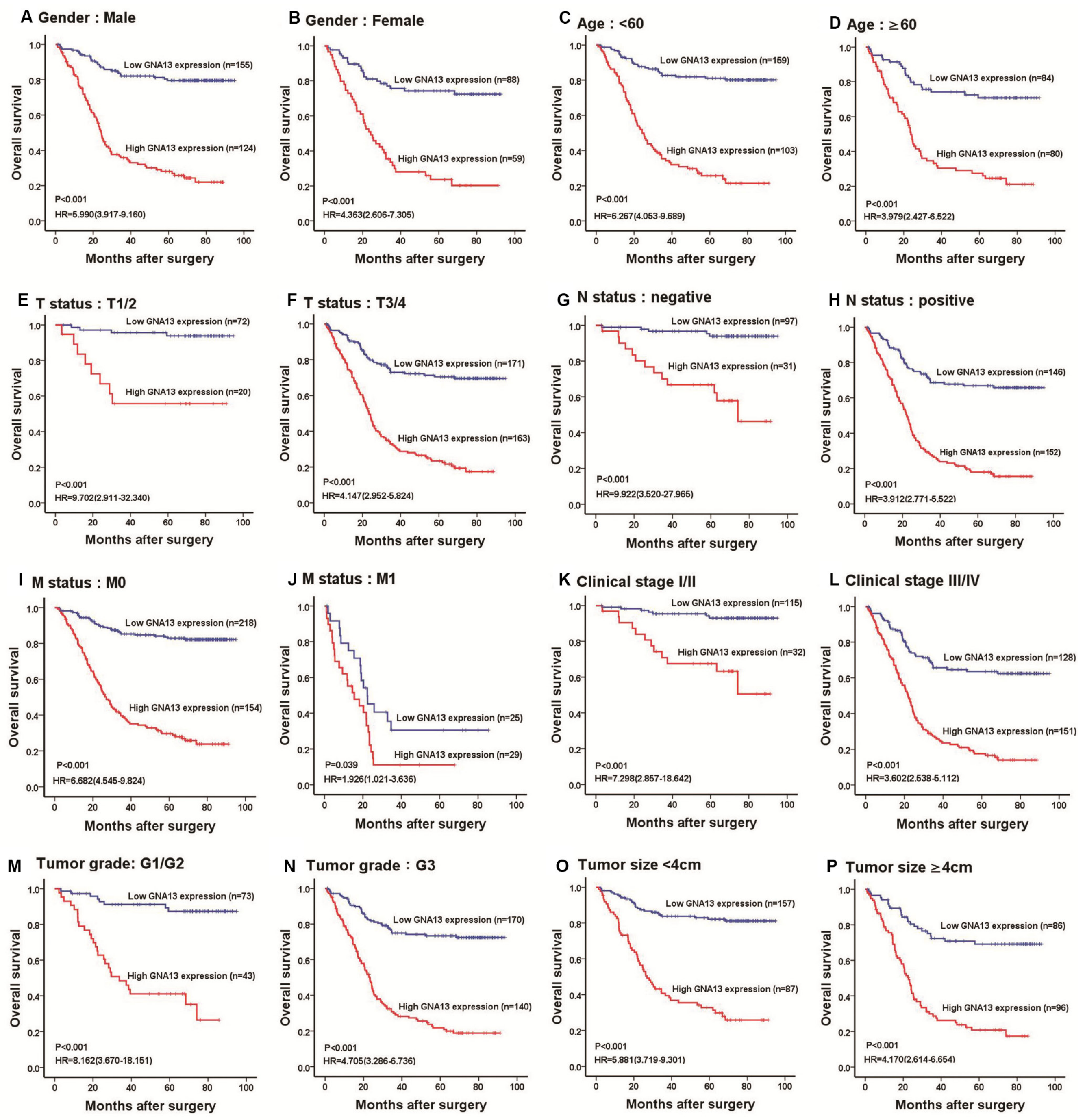

Supplementary Figure S3: Kaplan-Meier overall survival curves and log-rank test in GC patients stratified by Age (A and B), Gender (C and D), T statue (E and F), N statue (G and H), M statue (I and J), Clinical stage (K and L), Tumor grade (M and N), and Tumor size (O and P).

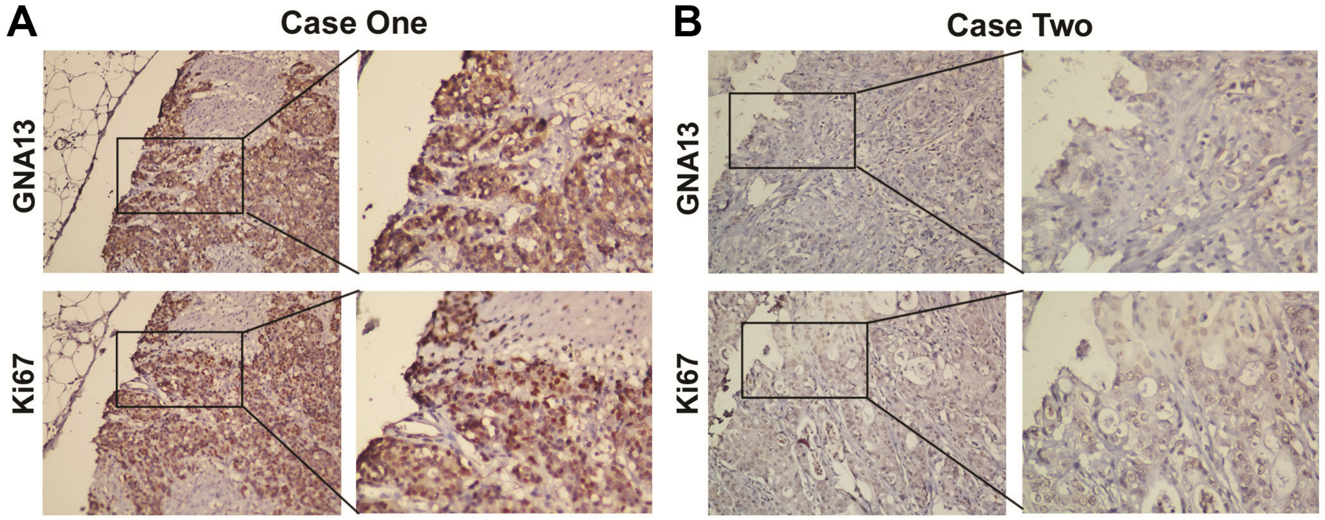

Supplementary Figure S4: GNA13 expression in gastric cancer tissues was positively associated with Ki-67 index. (**A**) High GNA13 IHC staining for GNA13 and Ki-67 were observed in the same GC tissues. (**B**) One GC sample showed both weak GNA13 and Ki-67 expression.

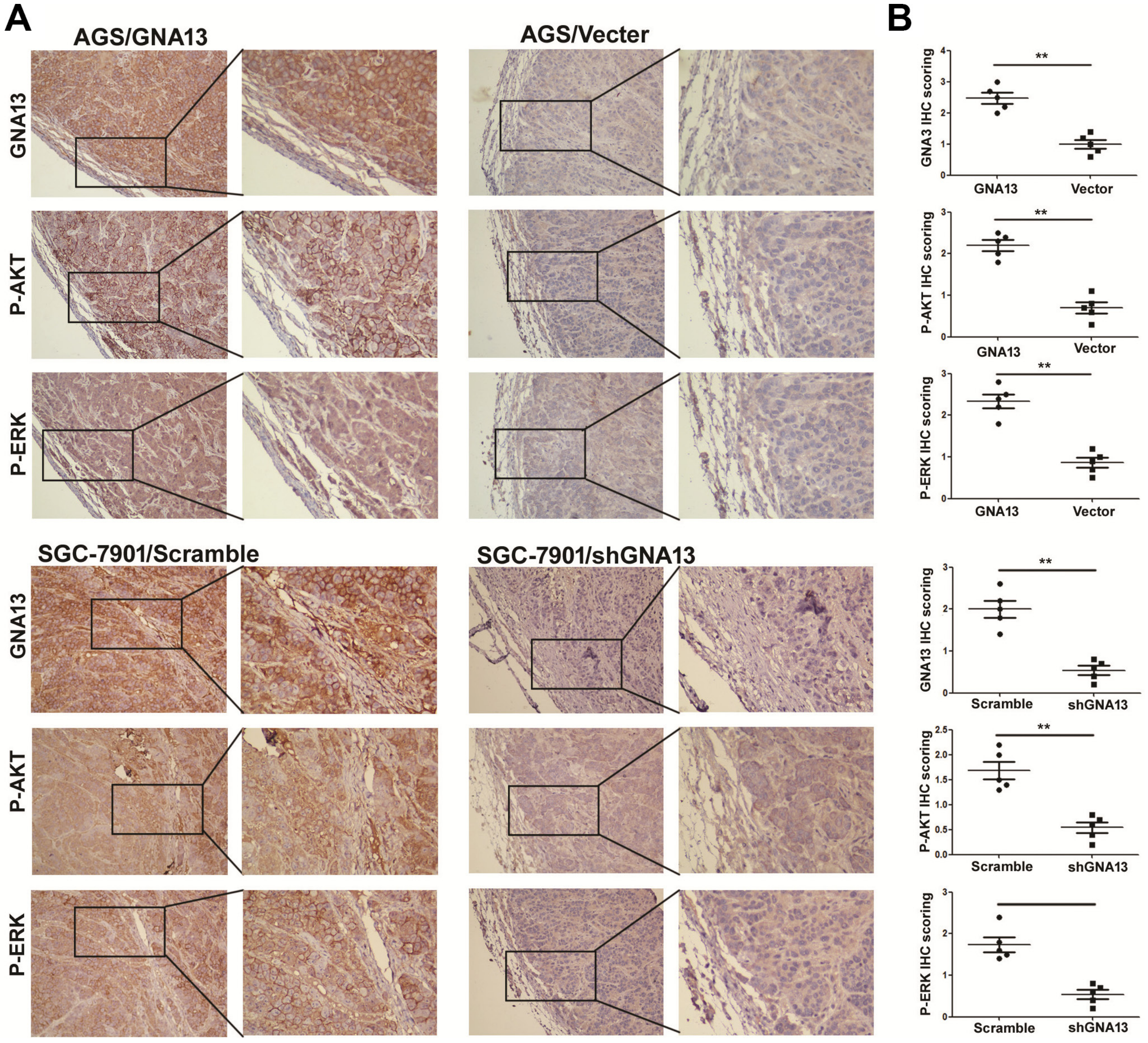

Supplementary Figure S5: The effects of GNA13 on the p-AKT and p-ERK protein levels *in vivo*. (**A**) The protein levels in GC tissues of the subcutaneous implantation nude mice models bearing GC were detected by IHC staining. (**B**) IHC scores for p-AKT and p-ERK were obviously increased in GNA13 transfected group, while decreased in GNA13 shRNA–infected cells. *$P < 0.05$; **$P < 0.01$.

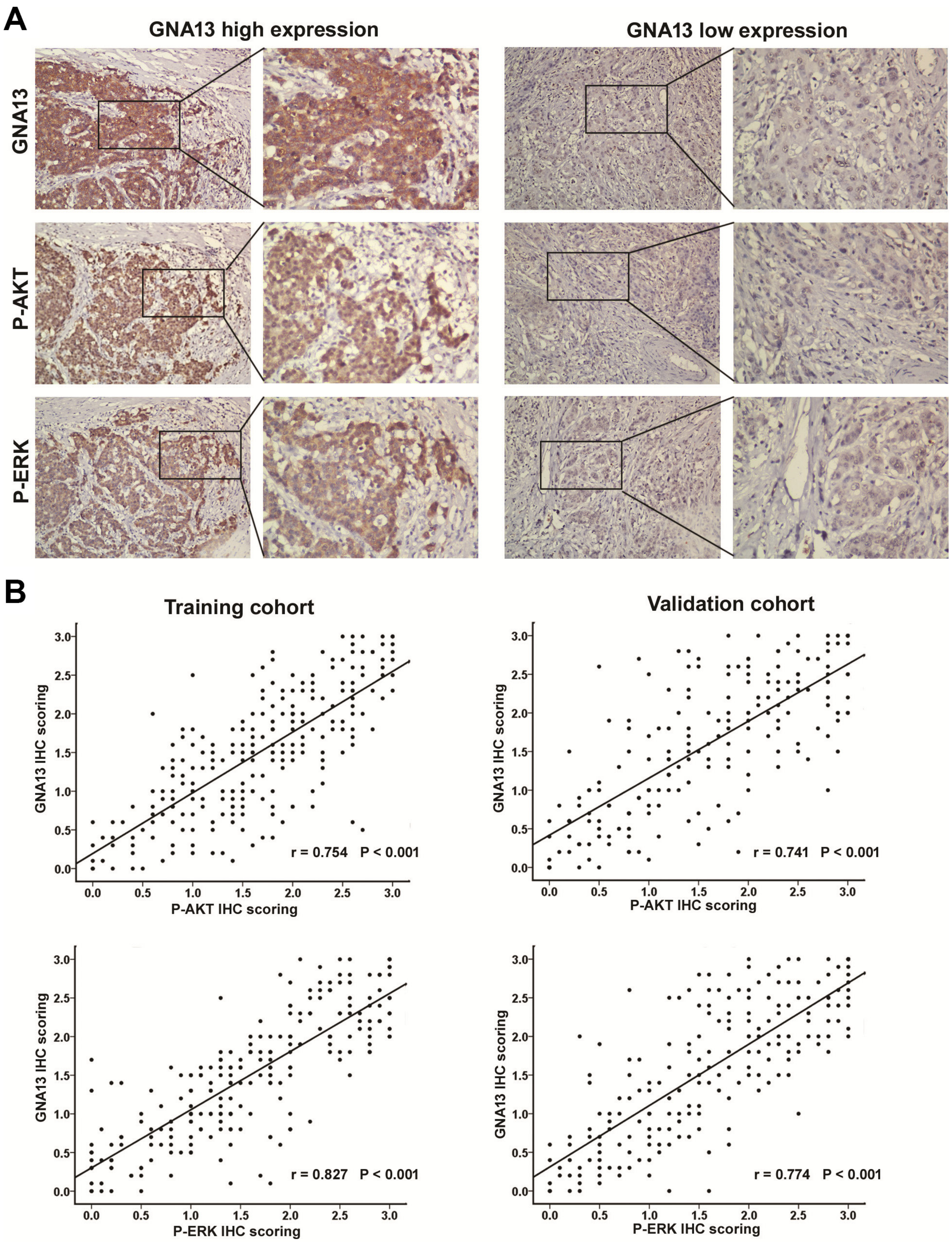

Supplementary Figure S6: The association of GNA13 and p-AKT, p-ERK in GC samples. (**A**) IHC Staining intensities for p-AKT and p-ERK were significantly reduced in low-GNA13 expressing (right) GC tissues than those with high-GNA13 levels (left). (**B**) The expression of GNA13 was significantly correlated with the expression of p-AKT and p-ERK in GC samples.

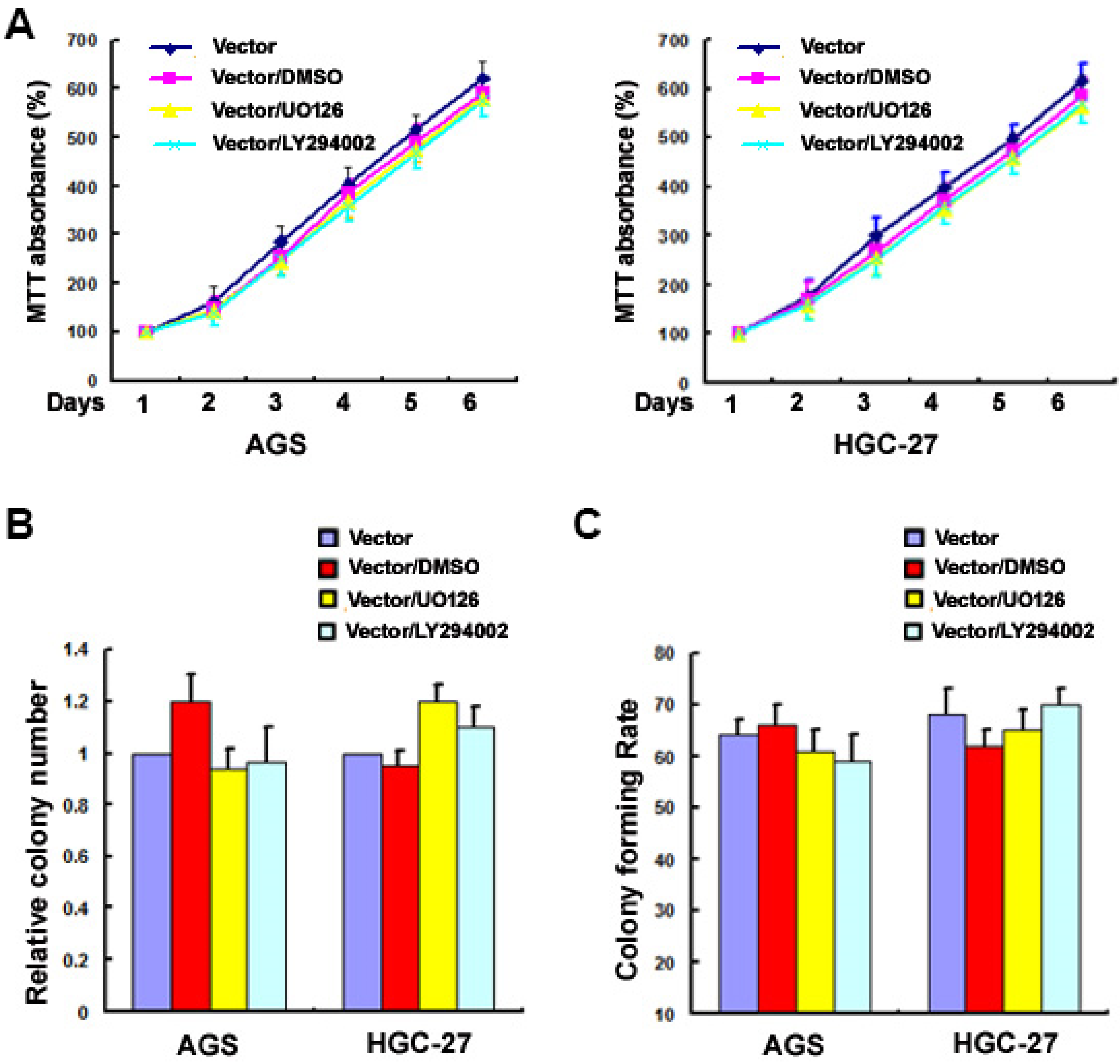

Supplementary Figure S7: The inhibition of PI3K/AKT and MAPK/ERK signaling pathway has no obvious effect on GC vector control cells. AGS/Vector and HGC-27/Vector proliferation and tumorigenicity were determined by MTT (**A**), colony formation assays (**B**) and anchorage-independent growth assay (**C**) after treatment with LY294002, U0126 or DMSO. $*P < 0.05$; $**P < 0.01$.

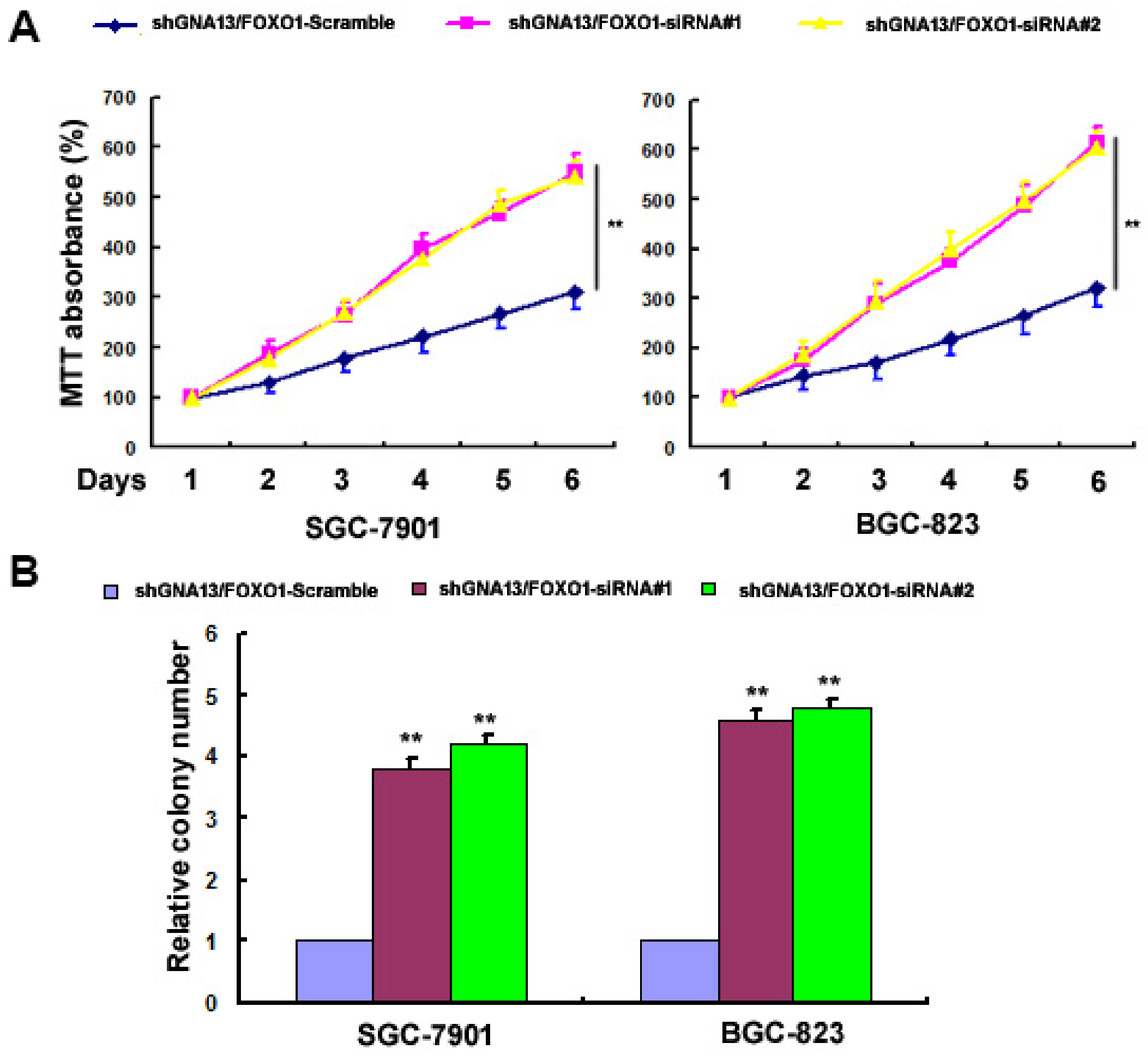

Supplementary Figure S8: Silencing FOXO1 increased the proliferation of GNA13 shRNA(s)–infected cells. Colony formation assays (**A**) and MTT assays (**B**) showed that silencing FOXO1 increased the proliferation of GNA13 shRNA(s)–infected GC cells.

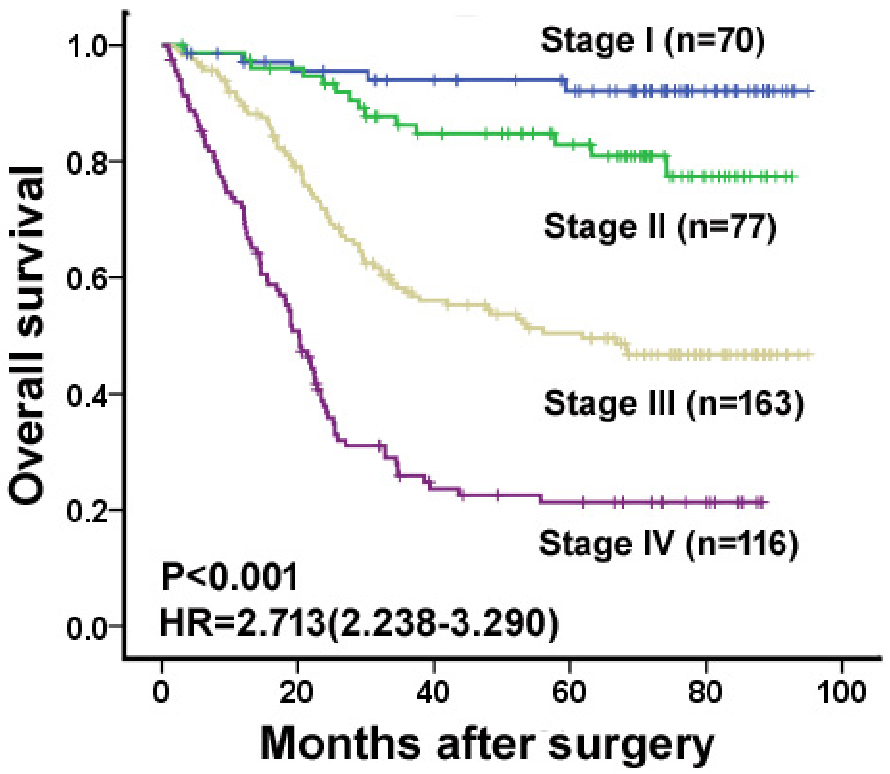

Supplementary Figure S9: Kaplan-Meier overall survival curves according to clinical stage.

Supplementary Table S1: Association of GNA13 expression with GC patients' clinicopathological features

Variables	GNA13 expression level							
	Training cohort				Validation cohort			
	Case	Low expression	High expression	*P* value	Case	Low expression	High expression	*P* value
Age		140	93			103	90	
< 60yr	149	95	54		113	64	49	
≥ 60yr	84	45	39	0.127	80	39	41	0.279
Gender								
Male	152	88	64		127	67	60	
Female	81	52	29	0.350	66	36	30	0.813
T statue								
T1/2	47	40	7		45	32	13	
T3/4	186	100	86	< 0.001	148	71	77	0.006
N								
N0	65	54	11		63	43	20	
N1/2	168	86	82	< 0.001	130	60	70	0.004
M								
M0	213	132	81		159	86	73	
M1	20	8	12	0.055	34	17	17	0.665
Clinical stage								
I/II	77	66	11		70	49	21	
III/IV	156	74	82	< 0.001	123	54	69	< 0.001
Grade								
G1/2	69	46	23		47	27	20	
G3	164	94	70	0.183	146	76	70	0.519
Tumor size								
< 4cm	120	82	38		124	75	49	
≥ 4cm	113	58	55	0.008	69	28	41	0.008
Ki-67 expression								
< 50%	135	122	13		116	87	29	
≥ 50%	98	18	80	< 0.001	77	16	61	< 0.001

GC, gastric cancer

Supplementary Table S2: Clinicopathological characteristics of 426 GC patients in training and validation cohort

	Number of cases (%)	
	Training cohort	Validation cohort
**Age**		
< 60yr	149 (63.9)	113 (58.5)
≥ 60yr	84 (36.1)	80 (41.6)
**Gender**		
Male	152 (65.2)	127 (65.8)
Female	81 (34.8)	66 (34.2)
**T statue**		
T1/2	47 (20.2)	45 (23.3)
T3/4	186 (79.8)	148 (76.7)
**N statue**		
N0	65 (27.9)	63 (32.6)
N1/2	168 (72.1)	130 (67.4)
**M statue**		
M0	213 (91.4)	159 (82.4)
M1	20 (8.6)	34 (17.6)
**Clinical stage**		
I/II	77 (33.0)	70 (36.3)
III/IV	156 (67.0)	123 (63.7)
**Grade**		
G1/2	69 (29.6)	47 (24.4)
G3	164 (70.4)	146 (75.6)
**Tumor size**		
< 4 cm	120 (51.5)	124 (64.2)
≥ 4 cm	113 (48.5)	69 (35.8)
**Vital status**		
Alive	128 (54.9)	115 (59.6)
Death	105 (45.1)	78 (40.4)
**Therapy**		
Surgery only	108 (45.1)	84 (43.5)
Surgery + CT	125 (54.9)	109 (56.5)

GC, gastric cancer; CT, chemotherapy

Supplementary Table S3: Sequences for primers used in this study

GNA13-OE-F1	TTATTGGTACCATGGCGGACTTCCTGCCGTC
GNA13-OE-R1	CTCACTCTAGAATCACTGTAGCATAAGCTGCT
GNA13-qPF	TCGGGAAAAGACCTATGTGAA
GNA13-qPR	CAACCAGCACCCTCATACCT
cyclinD1-qPF	TCTGTGCCACAGATGTGAAG
cyclinD1-qPR	AGCGTGTGAGGCGGTAGTAG
p21-qPF	GATGTGCCTATGGTCCTAGT
p21-qPR	CATCGTCAACACCCTGTCTT
p27-qPF	GCGGCAGAAGATTCTTCTTCG
p27-qPR	TGCTCCACAGTGCCAGCATT
Ki67-qPF	CATCAAGGAACAGCCTCAAC
Ki67-qPR	GTTGACTTCGGCTGATAGAC
GAPDH-qPF	CCCACATGGCCTCCAAGGAGTA
GAPDH-qPR	GTGTACATGGCAACTGTGAGGAGG
